# The efficacy and safety of glucokinase activators for the treatment of type-2 diabetes mellitus: A protocol for systematic review and meta-analysis

**DOI:** 10.1097/MD.0000000000024873

**Published:** 2021-02-19

**Authors:** Qian Gao, Wenjun Zhang, Tingting Li, Guojun Yang, Wei Zhu, Naijun Chen, Huawei Jin

**Affiliations:** Affiliated Hospital of Shaoxing University of Edocrine and Metabolism Department, Zhejiang, China.

**Keywords:** glucokinase activators, meta-analysis, type-2 diabetes mellitus

## Abstract

**Background:**

Glucokinase activators are a novel family of glucose-lowering agents used for the treatment of type-2 diabetes mellitus (T2DM). Glucokinase activators blind to GK activate the enzyme allosterically. Treatment with different GKAs has been shown to reduce fasting and postprandial glucose in patients with type 2 diabetes. We compared the efficacy/safety of glucokinase activators in T2DM patients through a meta-analysis.

**Methods:**

We searched PubMed, Excerpt Medica Database, and Cochrane Central Register of Controlled Trials databases for articles published before December 30, 2020. Two independent reviewers extracted the information from article. The quality of articles were assessed by 2 independent reviewers using the 5 items of scale proposed by Jadad. We computed the weighted mean difference and 95% confidence interval (CI) for a change from baseline to the study endpoint for glucokinase activators vs placebo. Egger test and Begg test were used to assess the possible publication bias caused by the tendency of published studies to be positive.

**Results:**

The present meta-analysis will compare the efficacy and safety of glucokinase activators and placebo for the treatment of T2DM.

**Conclusions:**

This meta-analysis will provide advanced evidence on the efficacy and safety of glucokinase activators for the treatment of T2DM.

**Ethics and dissemination:**

Ethical approval and patient consent are not required because this study is a literature-based study. This systematic review and meta-analysis will be published in a peer-reviewed journal

**PROSPERO registration number:**

CRD42021220364.

## 1 Introduction

Glucokinase activity (GKA) is associated with glucose-regulated insulin and glucagon sectretion in the pancreas, acting as a glucose sensor. In the liver, glucakinase (GK) processes glucose after a meal and coverts it into glycogen. Therefore, glucokinase has a central role in glucose homoeostasis and the blood glucose threshold was set at 4 to 6.5 mmol/L.^[1]^ The mutation of human GK gene causes the decrease of GK activity in β - cells and the increase of blood glucose threshold, which leads to moderate fasting hyperglycemia in Mody patients.^[2]^ It has been confirmed clinically that impaired GK can cause glucose metabolic diseases including the most common T2DM.^[3,4]^ GKA can effectively reduce glycosylated hemoglobin level and improve β-cell function in T2DM by improving GK function. The low affinity of GK to glucose makes it show activity at high glucose concentration, which indicates that GK may have a lower risk of hypoglycemia. However, in some early clinical trials, the incidence of hypoglycemia in the GKA drug piragliatin and mk-0941 experimental groups was not low.^[5,6]^ At the same time, in some long-term administration experiments, it was found that GKA drugs azd1656 and mk-0941 lost efficacy after several months of administration.^[5,6]^ However, the dozagliatin study of Hualing medicine, which has completed the phase III clinical trial, shows that the decrease of glycosylated hemoglobin can reach 1.12% after oral GKA twice a day.^[7,8]^ With the increase of dosage, the proportion of patients whose glycated hemoglobin (HbA1c) falls to the standard is also increasing, while hypoglycemia does not increase significantly. In order to provide new evidence-based medical evidence for clinical treatment, we undertook a meta-analysis to assess the efficacy and safety of GKAs in T2DM patients.

## 2 Methods

### 2.1 Study registration

This systematic review and meta-analysis protocol were registered in International Prospective Register of Systematic Reviews (PROSPERO) (registration no. CRD42021220364, https://www.crd.york.ac.uk/prospero/#recordDetails).

### 2.2 Search strategy

We conducted a search of PubMed, Excerpt Medica Database, the Cochrane Central Register of Controlled Trials databases for articles published before December 30, 2020 using the search terms “glucokinase activator,” “Dorzagliatin,” “HMS5552,” “Piragliatin,” “RO4389620,” “AMG 151,” “ARRY-403,” “AZD1656,” “AZD6370,” “TMG-123,” “MK-0941, “TTP-399,” “SY004,” “GKM001,” for randomized controlled trials on T2DM patients. The search strategy in detail is shown in Table 1.

**Table 1:** Search strategy used in this study.

Literature databases	Search items
Pubmed	(“Diabetes Mellitus, Type 2”[MeSH Terms] OR “Type 2 Diabetes Mellitus” OR “NIDDM” OR “Type 2 Diabetes”) AND (“glucokinase activator”[MeSH Terms] OR “Dorzagliatin” OR “HMS5552” OR “Piragliatin” OR “RO4389620” OR “AMG 151” OR “ARRY-403” OR “AZD1656” OR “AZD6370” OR “TMG-123” OR “MK-0941” OR “TTP-399” OR “SY004” OR “GKM001”) AND clinical trial[ptyp]
EMBASE	(“Diabetes Mellitus, Type 2”/exp OR “Type 2 Diabetes Mellitus” OR “NIDDM” OR “Type 2 Diabetes”) AND (“glucokinase activator”/exp OR “ Dorzagliatin”:ti, ab, kw OR “HMS5552”:ti, ab, kw OR“Piragliatin”:ti, ab, kw OR “RO4389620”:ti, ab, kw OR” AMG 151 “ti, ab, kw OR “ARRY-403”:ti, ab, kw OR “AZD1656”:ti, ab, kw OR “AZD6370”:ti, ab, kw OR “TMG-123”:ti, ab, kw OR “MK-0941”:ti, ab, kw OR “TTP-399”:ti, ab, kw OR “SY004”:ti, ab, kw OR “GKM001”:ti, ab, kw) AND ’randomized controlled trial’/de
CENTRAL	((Diabetes Mellitus, Type 2):ti, ab, kw OR (Type 2 Diabetes Mellitus):ti, ab, kw OR (NIDDM):ti, ab, kw OR (Type 2 Diabetes):ti, ab, kw) AND ((glucokinase activator):ti, ab, kw OR (Dorzagliatin):ti, ab, kw OR (HMS5552):ti, ab, kw OR (Piragliatin):ti, ab, kw OR (RO4389620):ti, ab, kw OR (AMG 151):ti, ab, kw OR (ARRY-403):ti, ab, kw OR (AZD1656):ti, ab, kw OR (AZD6370):ti, ab, kw OR (TMG-123):ti, ab, kw OR (MK-0941):ti, ab, kw OR (TTP-399):ti, ab, kw OR ("SY004):ti, ab, kw) OR (GKM001):ti, ab, kw

### 2.3 Study selection

Studies were eligible if they:

1. were randomized controlled trials;
2. compared a GAKs with a placebo;
3. treated patients for ≥4 weeks;
4. had at least 1 baseline and post-treatment efficacy and/or safety outcome of interest;
5. used T2DM patients aged ≥18 years;
6. were published in English.

### 2.4 Data extraction

**Figure 1:**
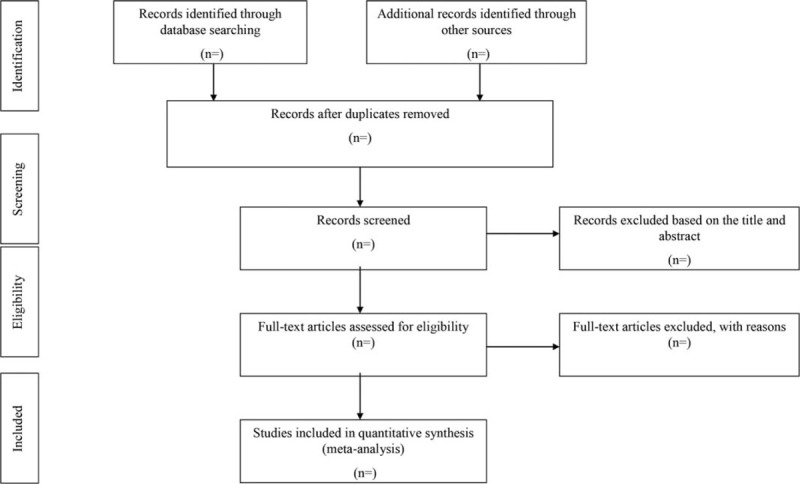
PRISMA flow chart.

As shown in Figure 1, the study selection was divided into 2 steps, which were completed by 2 researchers. Two independent reviewers extracted the following information from articles that met the inclusion criteria:

1. publication information (first author's name and year of publication);
2. baseline characteristics of the study (study size, participants’ age, dose for each arm, duration of follow-up, and country);
3. outcomes regarding efficacy and safety [change from baseline to the study endpoint for levels of HbA_1c_, fasting plasma glucose, postprandial blood glucose, homeostatic model assessment for β-cell function/insulin resistance (HOMA-β and HOMA-IR), body weight, hypoglycemia events, adverse events, serious adverse events].

If the same trial reported data at different follow-up durations, we extracted the data corresponding to the longest follow-up period. If a study reported results on the effects of GKAs at different doses, we extracted data corresponding to the effective doses for each GKAs.

### 2.5 Quality assessment

Articles meeting the inclusion criteria were assessed by 2 independent reviewers using the 5 items of scale proposed by Jadad for published studies that evaluate randomization (0–2 points), double-blinding (0–2 points), and description of withdrawals (1 point).^[9]^ Scores ranged from 0 to 5 and a score ≥3 indicated that a study was of “high quality.” The score was not used as a criterion for selection of a study; it was used only for descriptive purposes. Disagreements between the reviewers were discussed until a consensus was reached.

### 2.6 Statistical analyses

Continuous data were summarized as the weighted mean difference with 95% confidence intervals (CIs) for the change from baseline to the study endpoint for GKAs vs P. Dichotomous data were summarized as the risk difference (RD) with a 95% CI. If the 95% CI included a value of 0, we considered the difference between the GKAs and P to be not significant. Heterogeneity was assessed using the Q-statistic and *I*^*2*^ metric (*I*^*2*^ values of 25%, 50% and 75% were considered to indicate “low,” “medium,” and “high” heterogeneity, respectively) among trials. A *P-*value of the Q-statistic <.1 and *I*^*2*^ > 50% represented “substantial variability” and a random-effect model was used, otherwise, a fixed-effect model was used.^[10,11]^ To estimate a possible publication bias caused by the tendency of published studies to be positive, Egger test and Begg test were used and HbA_1c_ level was considered to be the main outcome variable.^[12]^ Sensitivity analysis was undertaken by omitting 1 study at a time and computing the pooled effect size of the remaining studies to evaluate if the results were affected markedly by a single study. All analyses were done using Stata v11.0 (Stata, College Station, TX). This meta-analysis was conducted according to the Preferred Reporting Items for Systematic Reviews and Meta-analyses (PRISMA) statement.^[13]^

## 3 Discussion

Mounting evidence implicates β-cell dysfunction as the primary defect associated with the progression of T2DM, and defective early phase insulin release has been clearly demonstrated in T2DM.^[14]^ Many diabetes patients have experienced frustrations from poor glycemic control despite adherence.^[15]^ There is still an urgent need for clinically differentiated oral antidiabetic agents to address drivers of β-cell dysfunction and repair the defective glucose sensor function. GKAs are a relatively new therapeutic class of oral anti-hyperglycemic drugs for T2DM. In order to provide new evidence-based medical evidence for clinical treatment, it is necessary to study the efficacy and safety of GAKs for the treatment of T2DM.

## 4 Conclusions

This meta-analysis will provide advanced evidence on the efficacy and safety of GKAs for the treatment of T2DM.

## Author contributions

**Conceptualization:** Qian Gao.

**Data curation:** Qian Gao, Wenjun Zhang.

**Investigation:** Tingting Li.

**Methodology:** Tingting Li.

**Resources:** Wei Zhu.

**Software:** Naijun Chen.

**Supervision:** Wenjun Zhang, Guojun Yang, Huawei Jin.

**Writing – original draft:** Qian Gao.

**Writing – review & editing:** Qian Gao.
